# A tribute to Graziella Berta (1948–2024): research milestones and highlights

**DOI:** 10.1007/s00572-024-01163-7

**Published:** 2024-08-01

**Authors:** Guido Lingua, Vivienne Gianinazzi-Pearson

**Affiliations:** 1 Dipartimento di Scienze e Innovazione Tecnologica, Università del Piemonte Orientale, Viale Teresa Michel, 11 - 15121 Alessandria, Italy; 2 SACCA SARL, 408 Avenue Josep Franch Clapers, 13210 Saint Rémy de Provence, France

**Keywords:** Graziella Berta, Arbuscular mycorrhizal fungi, Plant Growth-Promoting Bacteria, Root architecture, Root apical meristem, Polyploidy, Phytoremediation

## Abstract

Graziella Berta, a well-known mycorrhiza researcher, passed away in her home in Torino (Italy) on March 2nd, 2024, at the age of 75. We were both fortunate to know Graziella personally and to greatly appreciate her professionally, by working closely with her in the same research group in Alessandria (GL) or through many collaborative projects over the years (VGP). Here, we recall some of the milestones in her research and particularly the important contribution she has made to knowledge about plant interactions with mycorrhizal fungi and beneficial rhizosphere bacteria.

After graduating from Torino University in Natural Sciences with a thesis on the vegetation of the Italian Alps, Graziella Berta (Fig. 1) started her research career in that University in 1976 under the supervision first of Arturo Cerutti and later of Silvano Scannerini. She quickly developed a recognized expertise in microscopy, with a marked focus on ultrastructural studies. After her initial research on the septal pore apparatus in some fungal species, she became interested in the cellular organization of ericoid mycorrhiza and initiated an original line of research into the effects of mycorrhiza on host root development. Her work, which was particularly devoted to ericoid and arbuscular mycorrhiza (AM) at that time, revealed for the first time the effects of these symbioses on root apical meristem organization, root system morphology and architecture, host nuclear structure and activity, and on ploidy levels in colonized root cortex cells (Berta et al. 1990, 1995).

**Fig. 1:**
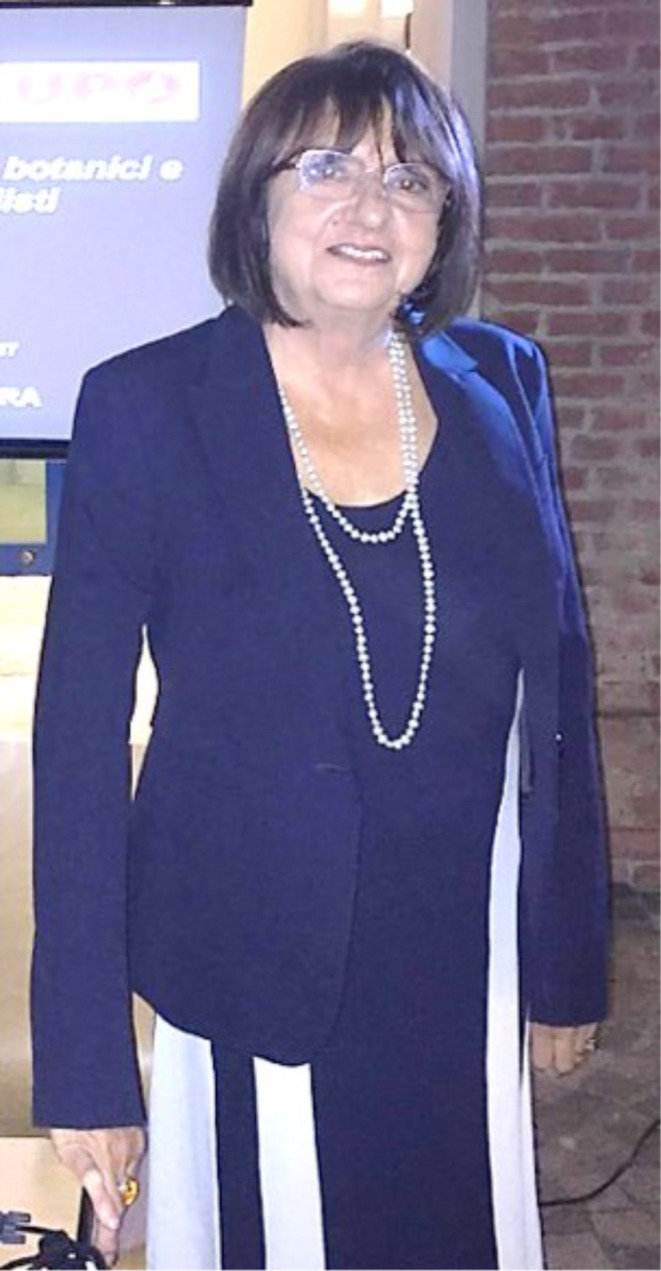
Graziella Berta in 2017, before giving a lecture

In 1995, after being appointed Full Professor in Botany, she moved to the Department of Science and Advanced Technology of the University of Torino in Alessandria, which became part of the Università del Piemonte Orientale “Amedeo Avogadro” in 1998. Graziella contributed to the inception, development and management of this newly founded institution, with many active roles among which President of the Degree in Biology, founder and Coordinator of the Ph.D. course in Environmental Sciences, Delegate of the Rector for Scientific Research, and first Director of the Dipartimento di Scienze e Innovazione Tecnologica.

In Alessandria, Graziella expanded her fundamental research on mycorrhiza to the effect of interactions between plant growth-promoting bacteria (PGPB) and AM fungi on plant growth and development, following a collaboration with Philippe Lemanceau (France) (Gamalero et al. 2002) and Bernard R. Glick (Canada). In particular, she developed a strong life-time interest in the cellular and molecular mechanisms involved in heavy metal stress resistance induced by PGPB and AM fungi in plants, and the use of these beneficial microbes for phytoremediation of polluted soils. Other lines of research for which she was known focused on how plant interactions with AM fungi and PGPB operate in mitigating pathogen infections, and on the important impact that these beneficial microbiota can have on the quality of plant products (Berta et al. 2014).

During her research career, at the start often in close interaction with Anna Fusconi, Graziella established close collaborations with Italian plant cytologists like Anna Maria Tagliasacchi, Sergio Sgorbati and Maria Maddalena Altamura, and with well-known european mycorrhizologists including Paola Bonfante, Manuela Giovannetti, Vivienne Gianinazzi-Pearson, Silvio Gianinazzi, John Hooker and David Atkinson. Her prolific research is reflected in an important publication record and her active participation to various EU projects and COST Actions, beyond being a regular attendee at ICOMs. All along her career she was very active in the Società Botanica Italiana (Italian Society of Botany), where she served as Coordinator of a workgroup on Cellular and Molecular Biology and as a member of the National Steering Committee.

Not only was Graziella a creative researcher, she also was a very enthusiastic teacher (Fig. 2), able to captivate her university students’ attention at any level, from introductory classes to Ph.D. seminars with lively lectures. Through her innate optimism and her scientific curiosity, she prompted her students to learn and explore new methods and techniques from any area of the biological sciences. At her retirement, in 2018, she was appointed Professor Emeritus and she kept actively collaborating with her department in research and teaching, a collaboration that she was forced to interrupt following the illness that eventually caused her death.

**Fig. 2:**
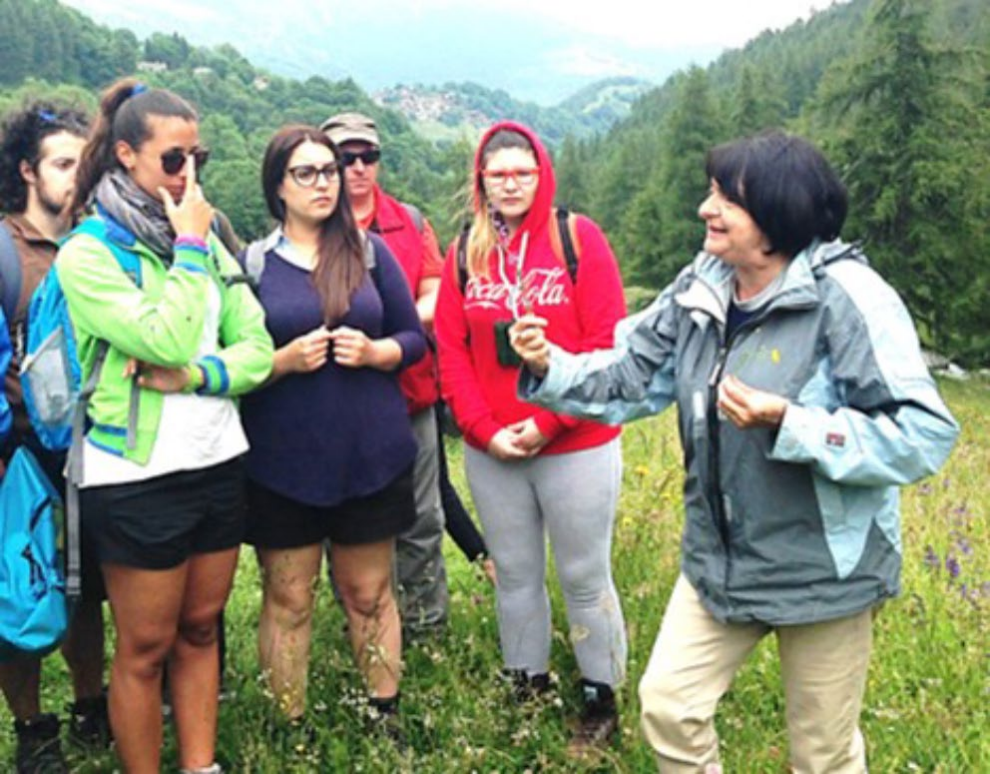
Graziella Berta (on the right) with her students, during a field excursion

Graziella was a cheerful, merry and very optimistic person, always ready to offer a second chance to everyone. She also was strongly determined in work and eager to participate in her scientific community. She loved the mountains and the sea, nature, classical music, sports, good food and company. Parallel to her career, Graziella also was a loving wife, mother, and recently grandmother. We express our closeness to her family Umberto, Francesca, Elena and the young Grace. She will be greatly missed by family, friends and colleagues.
